# Supplementary material for: Population variability under stressors is dependent on body mass growth and asymptotic body size
Source: R Soc Open Sci. 2020 Feb 26;7(2):192011. doi: 10.1098/rsos.192011 (PMC7062104; doi:10.1098/rsos.192011)
Supplement: Supplementary material [file rsos192011supp1.pdf]

Supplementary material for
Population variability under stressors is dependent on body mass growth and asymptotic body size.

Leonie Färber, Rob van Gemert, Øystein Langangen, Joël M. Durant, Ken H. Andersen

1 Methods

- Model

To initiate it we ran the model for 200 years, to allow reaching equilibrium. The stock numbers from year 200 were then the starting numbers for the scenario runs for a further 500 years.

Body mass growth rate A

A represents processes which gain and assimilate energy [1], and is derived from the von Bertalanffy growth equation [2]

Coming from the common length-based form of the von Bertalanffy growth $\frac{dl}{dt} = K(L_\infty - l)$, we can use K=3k [3] and $L_\infty = h^{-\frac{1}{3}}\frac{A}{k}$ [1] to have:

$$\frac{dl}{dt} = \frac{1}{3}\left(h^{-\frac{1}{3}}A - kl\right) \quad (1)$$

which can be then related to a weight based model with $w = hl^3$, where h is a coefficient in the weight length-relationship:

$$\frac{dw}{dt} = \frac{dhl^3}{dt} = h\frac{dl^3}{dl}\frac{dl}{dt} = 3hl^2\frac{dl}{dt} \quad (2)$$

This equation can then be rewritten to $\frac{dw}{dt} = Aw^n - kw$, with Aw^n represents energy gains and kw describes energy losses. Von Bertalanffy used in his calculations as exponent $n = \frac{2}{3}$ [2], where we will use $n = \frac{3}{4}$ [1, 4]

At some size, the losses are larger than the growth and growth stops, which is the asymptotic size $W_\infty = (\frac{A}{k})^{1/(1-n)}$. We rewrite the von Bertalanffy growth at age ($g(w_i)$) equation with:

$$g(w_i) = Aw_i{}^n(1 - (\frac{w_i}{W_\infty})^{1-n}) \qquad (3)$$

- Justification of the parameter values used

- *Metabolic scaling factor n: $n = \frac{3}{4}$ [4] because it acknowledges that the surface scales with weight > $\frac{2}{3}$, since it may be fractal [1, 5]*
- *Physiological mortality a: Based on calculations from [6] on natural mortality of fish and growth rate [1, 5].*
- *Start of fishing η_F: In many fish species, already juveniles are vulnerable to fishing gear (see e.g. [7, 8] as example for Atlantic cod and Atlantic herring) and thus we decided to have an entry level slightly before the maturity at $W_\infty 0.25$.*
- *Width of trawl-selectivity u_F: Our own choice, choosing a not too hard knife-edge transition, to allow for gradual shift in selectivity, but see sensitivity analysis (figure S5)*
- *Size at maturity η_m: From asymptotic length to length at maturity ratios [9] one gets ranges from around 0.4 to 0.8. When converted to weight as used here with $w = hl^3$,* where h is a coefficient in the weight length-relationship with *h=0.01 [10] we can approximate the value of W_∞0.25.*
- *Width of maturity switching function u_m: Our own choice, smoother than for the fishing switching function. See sensitivity analysis (figure S5)*
- *Recruitment efficiency ε_r: A value for recruitment efficiency is difficult to find in literature, we used an estimation from [11], which is based on calculations from n bioenergetics model and empirical measurements*
- *Egg weight w_{egg}: From data from FishBase [12] one can see that the geometric mean of teleosts egg weight stays consistently similar over the weight range and is around 1 mg (see e,g, figure 8.2 in [1]).*

- *Fraction of energy used for activity ε_a: Value derived from data from [13] and see figure 2a in [5].*
- *White noise standard deviation σ: Our own choice, to see large enough variability, but see sensitivity analysis (figure S5) (cf. [14])*
- *Red noise correlation coefficient c: Our own choice, however, in order to mimic large scale environmental drivers such as the NAO, we set the correlation high, here to 0.9, corresponding to a periodicity of around 10 years. But see also sensitivity analysis (figure S5).*

- Generating of the figures

For the figure 2 (main text) and figure A1 we plotted species within five families (Clupeidae, Gadidae, Scombridae, Sebastidae, and Engraulidae). We used the R package "*rfishbase*" Version 2.1.2 [15], containing data from the *FishBase* database [12]. With this package, we downloaded the families' body mass growth data for all the species. We extracted data where the hypothetical age at length at age 0 (t_0) is -1< t_0 >1 and where values were not classified as "doubtful". Instead of using K and asymptotic length, we used growth rate A and asymptotic size W_∞ parameters. K varies with asymptotic size, thus it is difficult to entangle directly from the parameter, if a species has slow or high growth or whether its asymptotic size is large or small [5].

Thus, from the von Bertalanffy growth parameter K, contained in the *FishBase* growth data, we calculated the growth parameter A [1]

$$A = 3 \cdot h^{0.25} \cdot \eta_m^{\left(-\frac{1}{12}\right)} \cdot K \cdot L_\infty^{3/4} \qquad (4)$$

Where h is the coefficient in the weight-length-relationship $W = hL^b$ and here used with h=0.01, as determined by Froese [10] with $log_{10}(h) = 4.544 - 2.174b$ and $b \approx 3$, which is approximately the median of coefficients of 1773 inspected species. This relationship is derived from juvenile growth and under the assumption that von Bertalanffy-growth and the growth rate calculated for this study lead to the same size at maturity.

Then we calculated the asymptotic weight from the asymptotic length L_∞ in the growth data

$$W_\infty = hL_\infty^3 \qquad (5)$$

We extracted the respective W_∞ and A from selected species from within these families, which are among the most fished species [16].

Clupeidae: *Sardinella* spp., *Clupea harengus*, *Sardina pilchardus*, *Brevoortia patronus*, *Sprattus sprattus*, *Sardinops melanostictus*

Gadidae: *Theragra chalcogramma*, *Gadus morhua*, *Micromesistius poutassou*

Scombridae: *Katsuwonus pelamis*, *Scomber japonicas*, *Scomber scombrus*, *Scomberomorus* spp., *Scomber colias*, *Rastrelliger kanagurta*, *Thunnus albacares*

Engraulidae: *Engraulis ringens*, *Engraulis japonicas*

In order to display slow growing species too, we chose the family of Sebastidae and selected *Sebastes* spp. We then computed a convex hull around these data points (figure S3, figure 2 main text).

All analyses were conducted in the R version 3.4.4 [17].

2 References

1 Andersen, K. H. 2019 *Fish Ecology, Evolution, and Exploitation-A New Theoretical Synthesis.*: Princeton University Press.
2 von Bertalanffy, L. 1957 Quantitative Laws in Metabolism and Growth. *Q Rev Biol*. **32**, 217-231.
3 von Bertalanffy, L. 1938 A quantitative theory of organic growth (Inquiries on growth laws. II). *Human Biol*. **10**, 181-213.
4 West, G. B., Brown, J. H., Enquist, B. J. 1997 A General Model for the Origin of Allometric Scaling Laws in Biology. *Science*. **276**, 122-126. (10.1126/science.276.5309.122)
5 Andersen, K. H., Beyer, J. E. 2015 Size structure, not metabolic scaling rules, determines fisheries reference points. *Fish Fish*. **16**, 1-22. (10.1111/faf.12042)
6 Gislason, H., Daan, N., Rice, J. C., Pope, J. G. 2010 Size, growth, temperature and the natural mortality of marine fish. *Fish Fish*. **11**, 149-158. (10.1111/j.1467-2979.2009.00350.x)
7 Hylen, A., Nakken, O., Nedreaas, K. 2008 *Northeast Arctic cod: fisheries, life history, stock fluctuations and management p. 83-118 In Norwegian spring-spawning herring and Northeast Arctic cod: 100 years of research and management*. Trondheim, Norway: Tapir Academic Press.
8 Dickey-Collas, M., Nash, R. D. M., Brunel, T., van Damme, C. J. G., Marshall, C. T., Payne, M. R., Corten, A., Geffen, A. J., Peck, M. A., Hatfield, E. M. C., *et al.* 2010 Lessons learned from stock collapse and recovery of North Sea herring: a review. *ICES J Mar Sci*. **67**, 1875-1886. (10.1093/icesjms/fsq033)

9 Beverton, R. J. H. 1992 Patterns of reproductive strategy parameters in some marine teleost fishes. *J Fish Biol*. **41**, 137-160. (10.1111/j.1095-8649.1992.tb03875.x)
10 Froese, R. 2006 Cube law, condition factor and weight–length relationships: history, meta-analysis and recommendations. *Journal of Applied Ichthyology*. **22**, 241-253. (10.1111/j.1439-0426.2006.00805.x)
11 Hartvig, M., Andersen, K. H., Beyer, J. E. 2011 Food web framework for size-structured populations. *J Theor Biol*. **272**, 113-122. (10.1016/j.jtbi.2010.12.006)
12 Froese, R., Pauly, D. *FishBase*. World Wide Web electronic publication. www.fishbase.org 2018.
13 Gunderson, D. R. 1997 Trade-off between reproductive effort and adult survival in oviparous and viviparous fishes. *Can J Fish Aquat Sci*. **54**, 990-998. (10.1139/f97-019)
14 Botsford, L. W., Holland, M. D., Field, J. C., Hastings, A. 2014 Cohort resonance: a significant component of fluctuations in recruitment, egg production, and catch of fished populations. *ICES J Mar Sci*. **71**, 2158-2170. (10.1093/icesjms/fsu063)
15 Boettiger, C., Lang, D. T., Wainwright, P. C. 2012 rfishbase: exploring, manipulating and visualizing FishBase data from R. *J Fish Biol*. **81**, 2030-2039. (10.1111/j.1095-8649.2012.03464.x)
16 FAO. 2018 The State of World Fisheries and Aquaculture 2018 - Meeting the sustainable development goals. Rome pp 227.
17 R Core Team. 2016 R: A language and environment for statistical computing. *R Foundation for Statistical Computing, Vienna, Austria*. URL http://www.R-project.org.

3 Figures

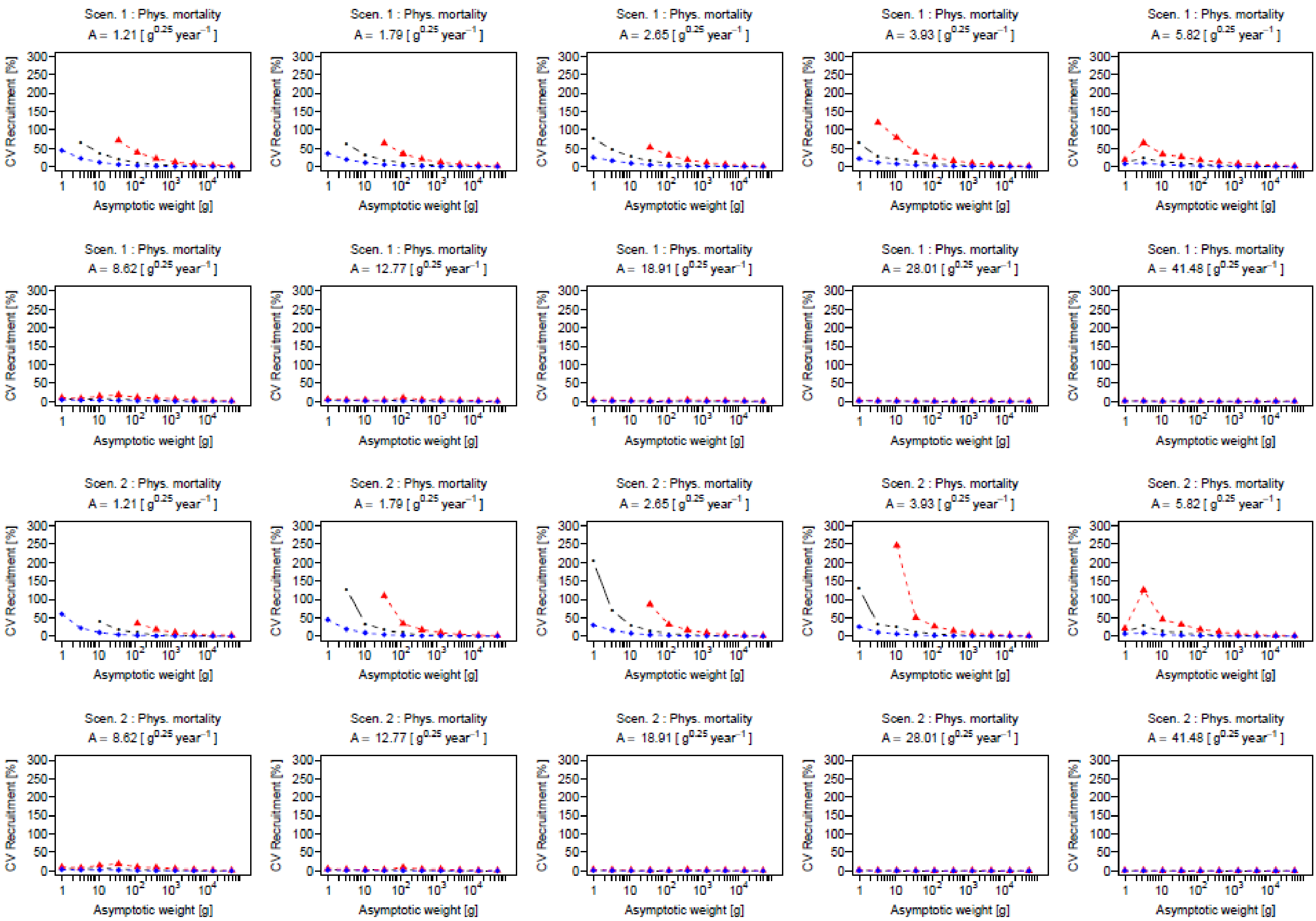

Figure S1 Sensitivity analysis of the parameters used in the model. The original parameter values in Table 2 in the main text were changed respectively by ± 20 %. The model was then run for 10 varying size classes and growth rates in the varying scenarios and the variability CV (here depicted in % variability) was calculated.

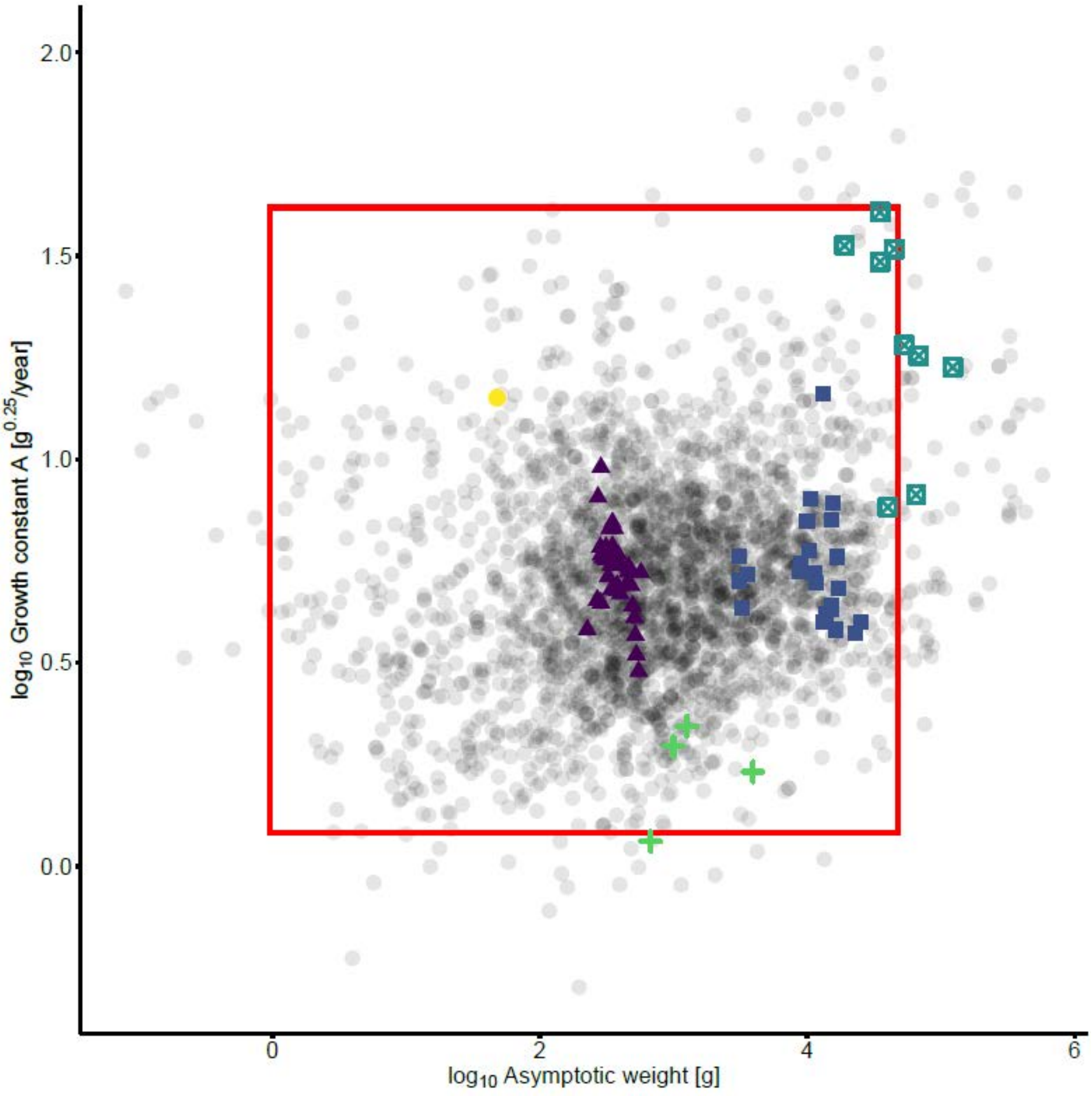

Figure S2 Visualization of the downloaded *Fishbase* [12] data, converted to asymptotic weight and our used growth constant (light grey dots). The yellow dot indicates the value for the anchovy, the triangles indicate data on Atlantic cod, squares indicate Atlantic herring, crosses indicate golden redfish and crosses squares yellowfin tuna values (colours (indicating belonging to a family and symbols as in figure A3). The selected range for our model is highlighted by a red square.

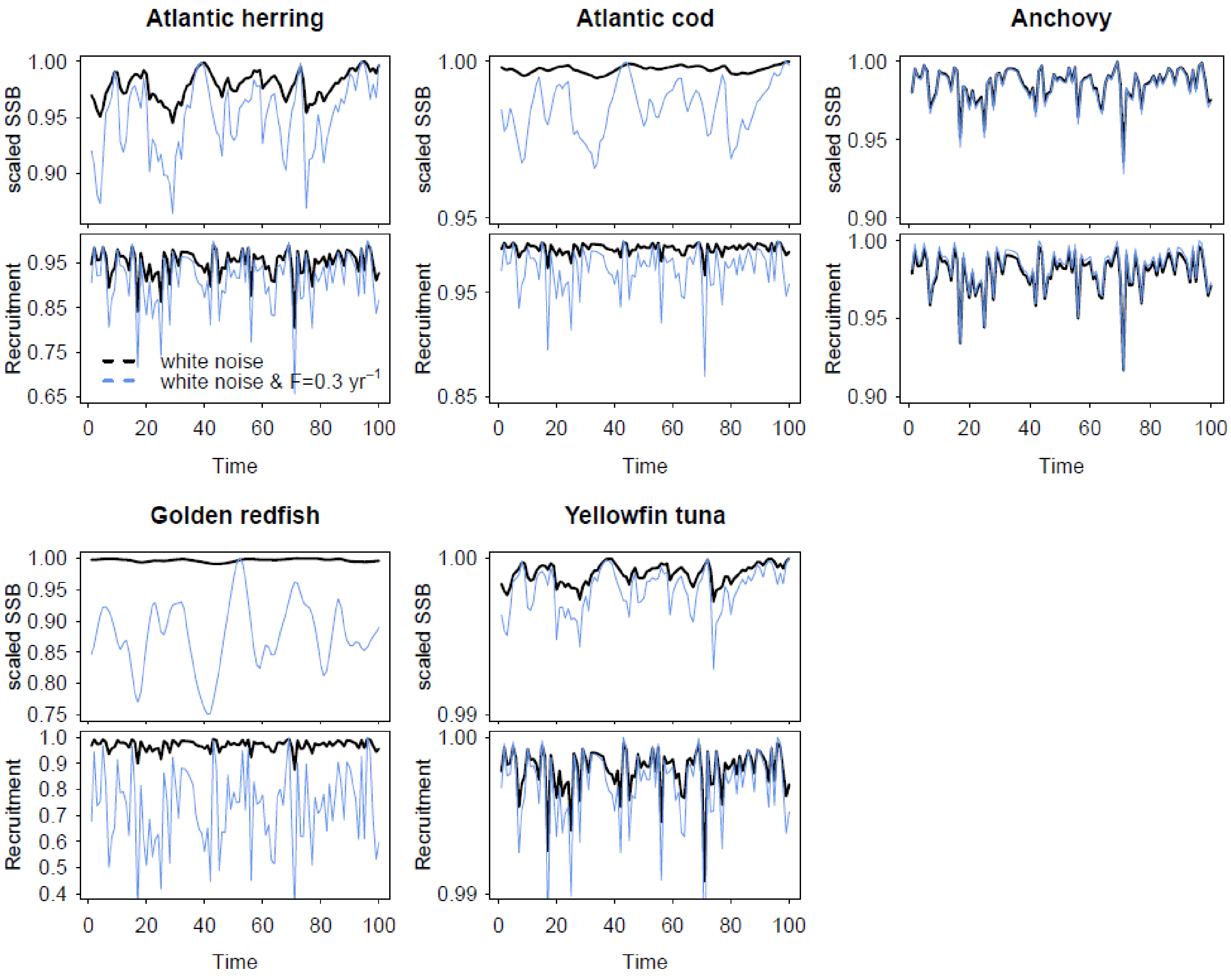

Figure S3 Visualization of variability in the spawning stock biomass (top panels) as well as recruitment (lower panels) for five selected species with varying life histories with white noise affecting recruitment efficiency and white noise in addition to 0.3 fishing mortality year^{-1}.

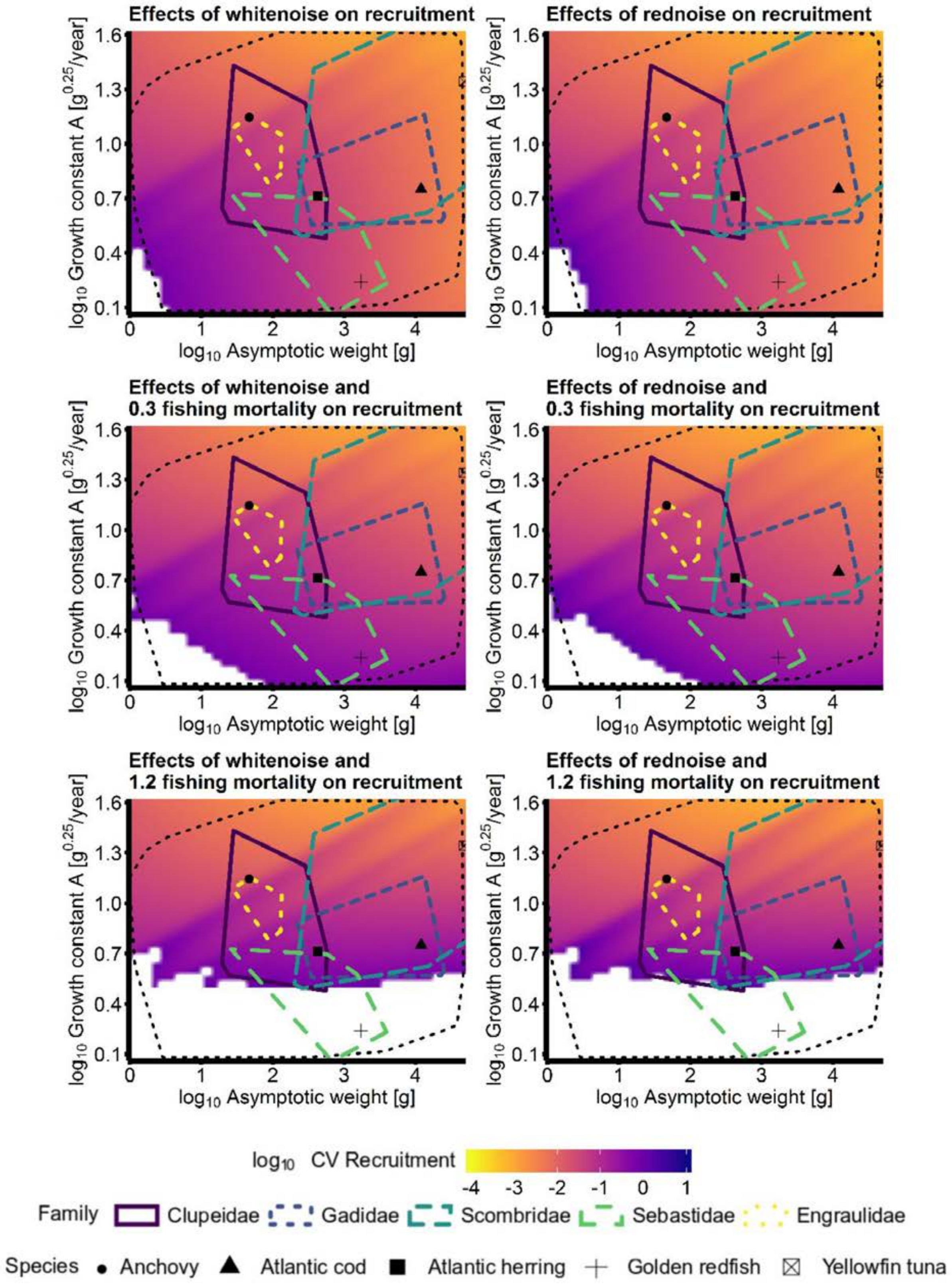

Figure S4 The logarithm with base 10 ($\log_{10}$) of the coefficient of variation (CV) as measure of variability over the whole parameter space of $\log_{10}$ size W_∞ and growth constant A for the scenario with white noise and 0.3 year^{-1} fishing mortality. The darker the colour, the higher the $\log_{10}$ CV and with that the variability. The white indicates that the stock collapsed. We

plotted the parameter space for species from five fish families, data obtained from the growth database contained in the R-package "*rfishbase*" and converted to W_∞ and A-values (Appendix). Five species of these families are indicated according to their position in the parameter space: Anchovy: $W_\infty \approx 50$ g, $A \approx 14$ $g^{0.25}$ $year^{-1}$; Atlantic cod: $W_\infty \approx 12$ kg, $A \approx 5.6$ $g^{0.25}$ $year^{-1}$; Atlantic herring: $W_\infty \approx 400$ g, $A \approx 5.2$ $g^{0.25}$ $year^{-1}$; golden redfish: $W_\infty \approx 1.7$ kg, $A \approx 1.7$ $g^{0.25}$ $year^{-1}$, and yellowfin tuna: $W_\infty \approx 48$ kg, $A \approx 22$ $g^{0.25}$ $year^{-1}$.

Around the available data from *FishBase* a convex hull was calculated (dotted black line) in order to indicate biological realistic parameter combinations.

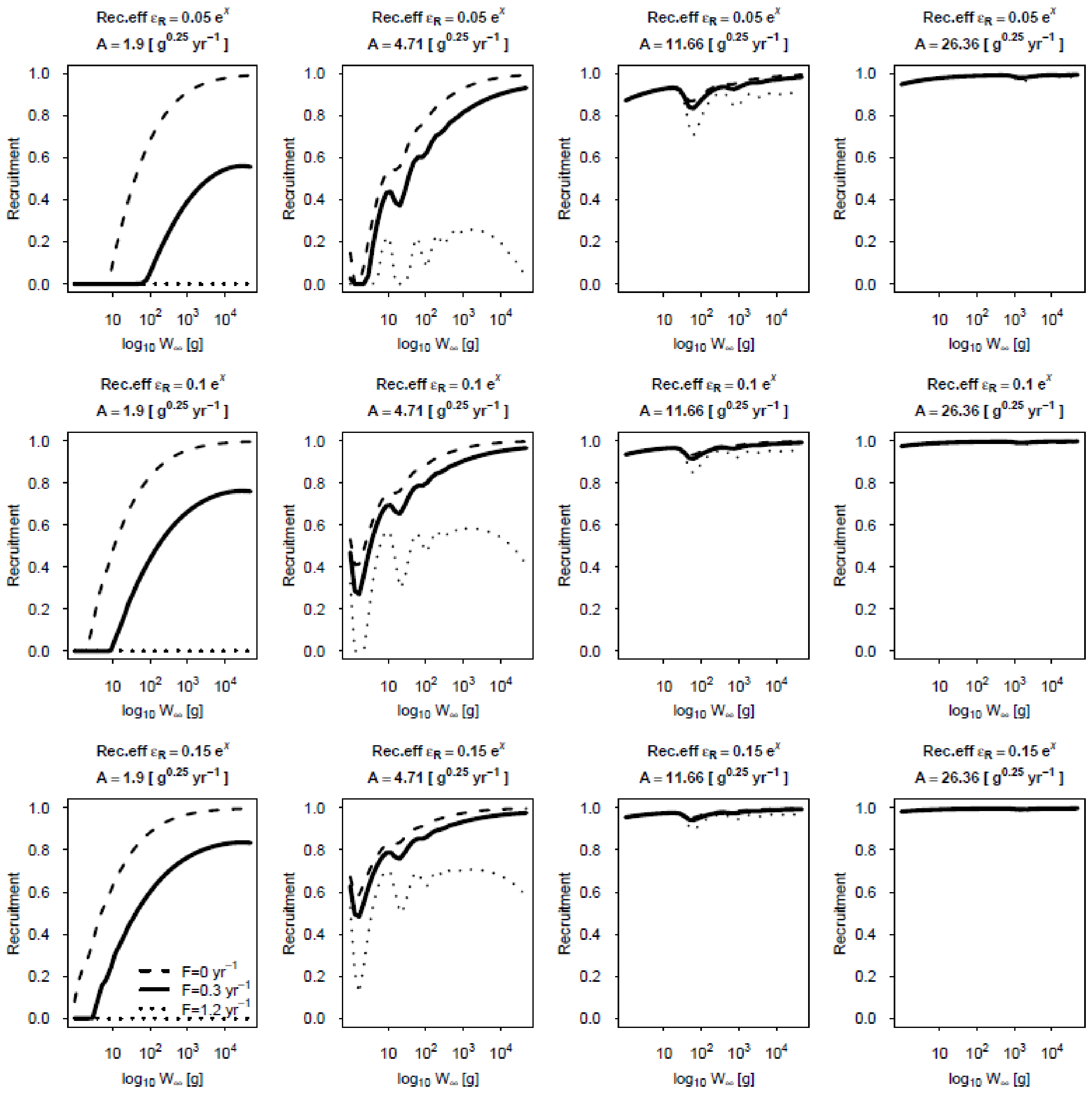

Figure S5 Recruitment over a wide size range and four different growth rates (columns): $A = 1.9$ $g^{0.25}year^{-1}$, $A = 4.71$ $g^{0.25}year^{-1}$, $A = 11.66$ $g^{0.25}year^{-1}$, and $A = 26.36$ $g^{0.25}year^{-1}$ (from slow to fast growth, left to right), and three different recruitment efficiencies ε_R (rows) (see Tables 1 & 2 main text): $\varepsilon_R = 0.05$, $\varepsilon_R = 0.1$, and $\varepsilon_R = 0.15$ (from low, to medium (used in our model) to high recruitment efficiency, top to bottom), which is affected by a lognormally distributed white noise x, leading to $\varepsilon_R e^x$. Three different fishing mortalities are applied: $F = 0$ year^{-1}, $F = 0.3$ year^{-1}, and $F = 1.2$ year^{-1} (dashed, solid, and dotted line, respectively). In the two columns on the right side, there is no strong effect of fishing detectable and the lines overlap.

4 R codes

```
#Parameters for the model

parms = list(
  m = 100, ####number of age classes
  wegg = 0.001, #Weight of egg, grams
  nm = 0.25, #size at maturity rel to maxw
  um = 10, #width of switching function in maturity #
  n = 3/4, #Exponent for consumption
  ea = 0.8, #Fraction of energy available for activity
  nF = 0.15, ##start of fishing rel. to maxw
  u = 3, #Sharpness of selection in trawl
  Fmax = 0, #Fishing mortality
  acon = 0.35, #Physiological mortality
  er = 0.1, #Recruitment efficiency
  Rmax = 1, #Maximum recruitment in SR relationship
  IT = 200, #number of iterations/time steps
  dt = 1, #length of time steps ###1 year
  correl = 0.9, #####correlation term for rednoise
  sd = 0.7 #Standard deviation of white noise
)

###equations

##maturity
matofun=function(w, maxw, parms){
  (1+(w/(parms$nm*maxw))^-parms$um)^-1
}

##von Bertalanffy growth at age
bertg<-function(maxw, A, parms){
  maxw*(1-exp(-(1-parms$n)*A*maxw^(parms$n-1)*ageclasses(parms)))^(1/(1-
parms$n)) ##bertalanffy growth, eq. B5.3 Fish and Fisheries p.42
}

#####age classes for matrix
ageclasses = function(parms){
  seq(1,parms$m, by=1)}

# ###SSB Biomass
SSB=function(num, w, maxw, parms){
  sum(c(matofun(w, maxw, parms)[1:(parms$m-1)]*num[1:(parms$m-
1)]*w[1:(parms$m-1)],0))
}

###trawlselectivity
trawlfun=function(w, maxw, parms){(1+(w/(parms$nF*maxw))^-parms$u)^-1}
##equation 5.3 in Andersen 2018 book

##Fishing mortality
fmor=function(w, maxw, parms){parms$Fmax*trawlfun(w, maxw, parms)}

####mortality
mort=function(A, w, maxw, parms){parms$acon*A*w^(parms$n-1)+fmor(w,maxw,
parms)}

###survival
survival<-function(A, w, maxw, parms){
  exp(-mort(A, w, maxw, parms)[c(1:(parms$m-1))])
```

```
}
###white noise, identical values in the same year for each run
f.whitenoise = function(parms){
  set.seed(123) #####to get always the same noise values
  noise = rnorm(parms$IT*parms$dt, mean=0, sd=parms$sd) ##random noise, new
noise for each new year

  #Extend noise vector so that it becomes equal in length to number of time
steps, but with same noise within a year
  noise = rep(noise, each = 1/parms$dt)
}

###red noise, identical values in the same year
f.rednoise = function(parms){
  rednoise=vector()
  whitenoise = f.whitenoise(parms)
  whitenoise = whitenoise[seq(1,by=1/parms$dt,length.out =
parms$dt*parms$IT)] #Reduce whitenoise vector back to one value per year
  rednoise[1]=whitenoise[1]
  for (i in 1:(parms$IT*parms$dt-1)){
    rednoise[i+1]=parms$correl*rednoise[i]+(1-
parms$correl^2)^(1/2)*whitenoise[i+1] ###equ. in Andersen book wrong
(p141)?, correlation of 1 not possible?, usage of another ref (but from a
lecture, need to find better one?!)
  }
  rednoise =  rep(rednoise, each = 1/parms$dt) #Extend rednoise vector,
keeping identical values within a single year
  return(rednoise)
}

###weight-specific egg production
eggpr=function(noise, A, maxw, parms){
  A*parms$er*exp(noise)*(1-parms$ea)*maxw^(parms$n-1)/parms$wegg
#######including the environmental noise term
}

###Recruitment, Beverton-Holt stock recruitment relationship
rec=function(noise,A,w,maxw,num,parms){
  parms$Rmax*eggpr(noise, A, maxw, parms)*(w[1]/parms$wegg)^(-
parms$acon)*SSB(num, w, maxw, parms)/
    (parms$Rmax + eggpr(noise, A, maxw, parms)*(w[1]/parms$wegg)^(-
parms$acon)*SSB(num, w, maxw, parms))
}
#
# ###Initial abundance
init.num = function(parms){
  seq(1,0,length=parms$m) #just some numbers to start with
}

###logarithmically distribution of growth rate A, 40 steps
dA<-(1.617836-0.08266683)/(40-1) ###values from second and fifth quantile
of density distribution fishbase data
A.seq<-c(10^0.08266683,10^(0.08266683+c(1:(40-1))*dA)) ###used in log
values

###logarithmically distribution of asymptotic size wmax for log.
distribution, 40 steps
dmax<-(4.679827-(-0.01697485))/(40-1)
maxw.seq<-c(10^(-0.01697485), 10^(-0.01697485+c(1:(40-1))*dmax))  ###values
from second and fifth quantile of density distribution fishbase data
```

```
### model function
run.model = function(noise,A,w,maxw,parms){
  surv.mat = matrix(0,nrow=parms$m,ncol=parms$m)
  num.track = matrix(0,nrow=parms$m,ncol=parms$IT) #Create matrix to track
abundance iterations in
  R.track = rep(0,  parms$IT)  #Create vector to track recruitment values
in
  SSB.track = rep(0,  parms$IT)  #Create vector to track SSB values in
  for(s in 2:parms$m){
    surv.mat[s,s-1]<-survival(A, w, maxw, parms)[s:s-1]
  }
  num.track[,1] <- surv.mat%*%init.num(parms) #Have the first column of the
abundance track matrix be equal to the initial abundance*survival matrix
  num.track[1,1]<-init.num(parms)[1]
  #Run the abundance iterations
  for(i in 2:(parms$IT)){
    #In each timestep, first calculate the abundance in the first weight
bin (Box 14, chapter 7, Ken's book)
    R.track[i-1] = rec(noise[i],A,w,maxw,num.track[,i-1],parms) #Store R
value
    num.track[,i] = surv.mat%*%num.track[,i-1] ###calculate numbers by
multiplying with survival matrix
    num.track[1,i]<-R.track[i-1]
    SSB.track[i-1] = SSB(num.track[,i], w, maxw, parms) #Store SSB value
  }
  return(list(num.track = num.track, R.track = R.track, SSB.track =
SSB.track))

}

####Scenarios for prerun with 200 timesteps (initiation)#####
##scenario 1: only whitenoise
###scenario 2: only rednoise
###scenario 3: whitenoise, 0.3 yr^(-1) fishing mortality
###scenario 4: rednoise, 0.3 yr^(-1) fishing mortality
###scenario 5: whitenoise, 1.2 yr^(-1) fishing mortality
###scenario 6: rednoise, 1.2 yr^(-1) fishing mortality


#######scenario 1####
noise = f.whitenoise(parms)
#Set fishing mortality
parms$Fmax = 0

scen1<-vector("list",length(maxw.seq))
for(t in 1:length(maxw.seq)){

  for(k in 1:length(A.seq)){
    w = bertg(maxw.seq[t], A.seq[k],parms)
    scen1[[t]][[k]]<-vector("list",length(A.seq))
    scen1[[t]][[k]] =
run.model(noise=noise,A=A.seq[k],w=w,maxw=maxw.seq[t],parms)
  }
}

#######model run scenario 2#######
noise = f.rednoise(parms)
#Set fishing mortality
parms$Fmax = 0
```

```
scen2<-vector("list",length(maxw.seq))
for(t in 1:length(maxw.seq)){

  for(k in 1:length(A.seq)){
    w = bertg(maxw.seq[t], A.seq[k],parms)
    scen2[[t]][[k]]<-vector("list",length(A.seq))
    scen2[[t]][[k]] =
run.model(noise=noise,A=A.seq[k],w=w,maxw=maxw.seq[t],parms)
  }
}

#######model runs scenario 3##########
noise = f.whitenoise(parms)
#Set fishing mortality
parms$Fmax = 0.3

scen3<-vector("list",length(maxw.seq))
for(t in 1:length(maxw.seq)){

  for(k in 1:length(A.seq)){
    w = bertg(maxw.seq[t], A.seq[k],parms)
    scen3[[t]][[k]]<-vector("list",length(A.seq))
    scen3[[t]][[k]] =
run.model(noise=noise,A=A.seq[k],w=w,maxw=maxw.seq[t],parms)

  }

}
#######model runs scenario 4##########
noise = f.rednoise(parms)

#Set fishing mortality
parms$Fmax = 0.3

scen4<-vector("list",length(maxw.seq))
for(t in 1:length(maxw.seq)){

  for(k in 1:length(A.seq)){
    w = bertg(maxw.seq[t], A.seq[k],parms)
   scen4[[t]][[k]]<-vector("list",length(A.seq))
    scen4[[t]][[k]] =
run.model(noise=noise,A=A.seq[k],w=w,maxw=maxw.seq[t],parms)

  }
}
#######model runs scenario 5##########
noise = f.whitenoise(parms)

#Set fishing mortality
parms$Fmax = 1.2

scen5<-vector("list",length(maxw.seq))
for(t in 1:length(maxw.seq)){

  for(k in 1:length(A.seq)){
    w = bertg(maxw.seq[t], A.seq[k],parms)
    scen5[[t]][[k]]<-vector("list",length(A.seq))
    scen5[[t]][[k]] =
run.model(noise=noise,A=A.seq[k],w=w,maxw=maxw.seq[t],parms)
  }
```

```
}


#######model runs scenario 6##########
noise = f.rednoise(parms)

#Set fishing mortality
parms$Fmax = 1.2

scen6<-vector("list",length(maxw.seq))
for(t in 1:length(maxw.seq)){

  for(k in 1:length(A.seq)){
    w = bertg(maxw.seq[t], A.seq[k],parms)
    scen6[[t]][[k]]<-vector("list",length(A.seq))
    scen6[[t]][[k]] =
run.model(noise=noise,A=A.seq[k],w=w,maxw=maxw.seq[t],parms)
  }
}

####Scenarios for prerun with 500 timesteps (initiation)#####
##use abundances from year 200 for inital numbers

##new iteration
parms$IT<-500


###run model with new starting numbers
run.model = function(noise,A,w,maxw,parms, numbers){
  surv.mat = matrix(0,nrow=parms$m,ncol=parms$m)
  num.track = matrix(0,nrow=parms$m,ncol=parms$IT) #Create matrix to track
abundance iterations in
  R.track = rep(0,  parms$IT)  #Create vector to track recruitment values
in
  SSB.track = rep(0,  parms$IT)  #Create vector to track SSB values in
  for(s in 2:parms$m){
    surv.mat[s,s-1]<-survival(A, w, maxw, parms)[s:s-1]
  }
  num.track[,1] <- surv.mat%*%numbers #Have the first column of the
abundance track matrix be equal to the initial abundance*survival matrix
  num.track[1,1]<-numbers[1]
  #Run the abundance iterations
  for(i in 2:(parms$IT)){
    #In each timestep, first calculate the abundance in the first weight
bin (Box 14, chapter 7, Ken's book)
    R.track[i-1] = rec(noise[i],A,w,maxw,num.track[,i-1],parms) #Store R
value
    num.track[,i] = surv.mat%*%num.track[,i-1] ###calculate numbers by
multiplying with survival matrix
    num.track[1,i]<-R.track[i-1]
    SSB.track[i-1] = SSB(num.track[,i], w, maxw, parms) #Store SSB value

  }
  return(list(num.track = num.track, R.track = R.track, SSB.track =
SSB.track))

}

###scenarios same as before
###scenario 1###
noise = f.whitenoise(parms)
```

```
#Set fishing mortality
parms$Fmax = 0

scen1<-vector("list",length(maxw.seq))
for(t in 1:length(maxw.seq)){

  for(k in 1:length(A.seq)){
    numbers<-scen1start[[t]][[k]]$num.track[,200]
    w = bertg(maxw.seq[t], A.seq[k],parms)
   scen1[[t]][[k]]<-vector("list",length(A.seq))
    scen1[[t]][[k]] =
run.model(noise=noise,A=A.seq[k],w=w,maxw=maxw.seq[t],parms, numbers)

  }

}

#######model runs scenario 2#######
noise = f.rednoise(parms)

#Set fishing mortality
parms$Fmax = 0

scen2<-vector("list",length(maxw.seq))
for(t in 1:length(maxw.seq)){

  for(k in 1:length(A.seq)){
    numbers<-scen2start[[t]][[k]]$num.track[,200]
    w = bertg(maxw.seq[t], A.seq[k],parms)
    scen2[[t]][[k]]<-vector("list",length(A.seq))
    scen2[[t]][[k]] =
run.model(noise=noise,A=A.seq[k],w=w,maxw=maxw.seq[t],parms, numbers)

  }

}


#######model runs scenario 3##########
noise = f.whitenoise(parms)

#Set fishing mortality
parms$Fmax = 0.3
scen3<-vector("list",length(maxw.seq))
for(t in 1:length(maxw.seq)){

  for(k in 1:length(A.seq)){
    numbers<-scen3start[[t]][[k]]$num.track[,200]
    w = bertg(maxw.seq[t], A.seq[k],parms)
    scen3[[t]][[k]]<-vector("list",length(A.seq))
    scen3[[t]][[k]] =
run.model(noise=noise,A=A.seq[k],w=w,maxw=maxw.seq[t],parms, numbers)

  }

}

#######model runs scenario 4##########
noise = f.rednoise(parms)
```

```
#Set fishing mortality
parms$Fmax = 0.3

scen4<-vector("list",length(maxw.seq))
for(t in 1:length(maxw.seq)){

  for(k in 1:length(A.seq)){
    numbers<-scen4start[[t]][[k]]$num.track[,200]
    w = bertg(maxw.seq[t], A.seq[k],parms)
    # maxi<-which(round(w,5)==maxw.seq[t])
    # w[maxi[2:length(maxi)]]<-0
    scen4[[t]][[k]]<-vector("list",length(A.seq))
    scen4[[t]][[k]] =
run.model(noise=noise,A=A.seq[k],w=w,maxw=maxw.seq[t],parms, numbers)

  }

}

#######model runs scenario 5##########
noise = f.whitenoise(parms)

#Set fishing mortality
parms$Fmax = 1.2

scen5<-vector("list",length(maxw.seq))
for(t in 1:length(maxw.seq)){

  for(k in 1:length(A.seq)){
    numbers<-scen5start[[t]][[k]]$num.track[,200]
    w = bertg(maxw.seq[t], A.seq[k],parms)
    scen5[[t]][[k]]<-vector("list",length(A.seq))
    scen5[[t]][[k]] =
run.model(noise=noise,A=A.seq[k],w=w,maxw=maxw.seq[t],parms, numbers)
  }

}

#######model runs scenario 6##########
noise = f.rednoise(parms)

#Set fishing mortality
parms$Fmax = 1.2

scen6<-vector("list",length(maxw.seq))
for(t in 1:length(maxw.seq)){

  for(k in 1:length(A.seq)){
    numbers<-scen6start[[t]][[k]]$num.track[,200]
    w = bertg(maxw.seq[t], A.seq[k],parms)
    scen6[[t]][[k]]<-vector("list",length(A.seq))
    scen6[[t]][[k]] =
run.model(noise=noise,A=A.seq[k],w=w,maxw=maxw.seq[t],parms, numbers)

  }
}


####calculate coefficient of variation for recruitment variability####
##########calculate recruitment variability######
```

```
Drec1<-Drec2<-Drec3<-Drec4<-Drec5<-Drec6<-list()
for(j in 1:length(maxw.seq)) { ###j is for all the maxw possibilities
  Drec1[[j]]<-Drec2[[j]]<-Drec3[[j]]<-Drec4[[j]]<-Drec5[[j]]<-Drec6[[j]]<-
array(NA)
  for (i in 1: 40){ ###i is for all the A possibilities
    Drec1[[j]][i]<-(sd(round(scen1[[j]][[i]]$R.track[1:499],4))/

ifelse(round(mean(scen1[[j]][[i]]$R.track[1:499]),2)>0.01,round(mean(scen1[
[j]][[i]]$R.track[1:499]),2),NA)) #####round, so numbers close to 0 do not
increase the variability
    Drec2[[j]][i]<-(sd(round(scen2[[j]][[i]]$R.track[1:499],4))/

ifelse(round(mean(scen2[[j]][[i]]$R.track[1:499]),2)>0.01,round(mean(scen2[
[j]][[i]]$R.track[1:499]),2),NA)) ##rednoise no fishing

    Drec3[[j]][i]<-(sd(round(scen3[[j]][[i]]$R.track[1:499],4))/

ifelse(round(mean(scen3[[j]][[i]]$R.track[1:499]),2)>0.01,round(mean(scen3[
[j]][[i]]$R.track[1:499]),2),NA))##whitenoise and 0.3 fishing

    Drec4[[j]][i]<-(sd(round(scen4[[j]][[i]]$R.track[1:499],4))/

ifelse(round(mean(scen4[[j]][[i]]$R.track[1:499]),2)>0.01,round(mean(scen4[
[j]][[i]]$R.track[1:499]),2),NA))##rednoise and 0.3 fishing

    Drec5[[j]][i]<-(sd(round(scen5[[j]][[i]]$R.track[1:499],4))/

ifelse(round(mean(scen5[[j]][[i]]$R.track[1:499]),2)>0.01,round(mean(scen5[
[j]][[i]]$R.track[1:499]),2),NA)) ##whitenoise and 1.2 fishing

    Drec6[[j]][i]<-(sd(round(scen6[[j]][[i]]$R.track[1:499],4))/

ifelse(round(mean(scen6[[j]][[i]]$R.track[1:499]),2)>0.01,round(mean(scen6[
[j]][[i]]$R.track[1:499]),2),NA))##rednoise and 1.2 fishing


  }
}

###dataframe for plotting
alld<-data.frame(Drec1=Drec1[[1]], Drec2=Drec2[[1]],  Drec3=Drec3[[1]],
                 Drec4=Drec4[[1]], Drec5=Drec5[[1]], Drec6=Drec6[[1]],
maxw=rep(maxw.seq[1], 40), A=A.seq)


for(j in 2:40){
  alld<-rbind(alld, list(Drec1[[j]], Drec2[[j]], Drec3[[j]], Drec4[[j]],
                         Drec5[[j]], Drec6[[j]], rep(maxw.seq[j], 40),
A.seq))
}
```
